# Transcriptomic Analysis of *Streptomyces coelicolor* Differentiation in Solid Sporulating Cultures: First Compartmentalized and Second Multinucleated Mycelia Have Different and Distinctive Transcriptomes

**DOI:** 10.1371/journal.pone.0060665

**Published:** 2013-03-28

**Authors:** Paula Yagüe, Antonio Rodríguez-García, María T. López-García, Juan F. Martín, Beatriz Rioseras, Jesús Sánchez, Angel Manteca

**Affiliations:** 1 Área de Microbiología, Departamento de Biología Funcional and Instituto Universitario de Biotecnología de Asturias (IUBA), Universidad de Oviedo, Oviedo, Spain; 2 Instituto de Biotecnología de León (INBIOTEC), León, Spain; University Paris South, France

## Abstract

Streptomycetes are very important industrial bacteria, which produce two thirds of all clinically relevant secondary metabolites. They have a complex developmental-cycle in which an early compartmentalized mycelium (MI) differentiates to a multinucleated mycelium (MII) that grows inside the culture medium (substrate mycelium) until it starts to growth into the air (aerial mycelium) and ends up forming spores. *Streptomyces* developmental studies have focused mainly on the later stages of MII differentiation (aerial mycelium and sporulation), with regulation of pre-sporulation stages (MI/MII transition) essentially unknown. This work represents the first study of the *Streptomyces* MI transcriptome, analyzing how it differs from the MII transcriptome. We have used a very conservative experimental approach to fractionate MI from MII and quantify gene expressions. The expression of well characterized key developmental/metabolic genes involved in bioactive compound production (actinorhodin, undecylprodigiosin, calcium-dependent antibiotic, cpk, geosmin) or hydrophobic cover formation-sporulation (*bld*, *whi*, *wbl*, *rdl*, *chp*, *ram*) was correlated with MII differentiation. Additionally, 122 genes conserved in the *Streptomyces* genus, whose biological function had not been previously characterized, were found to be differentially expressed (more than 4-fold) in MI or MII. These genes encoded for putative regulatory proteins (transcriptional regulators, kinases), as well as hypothetical proteins. Knowledge about differences between the MI (vegetative) and MII (reproductive) transcriptomes represents a huge advance in *Streptomyces* biology that will make future experiments possible aimed at characterizing the biochemical pathways controlling pre-sporulation developmental stages and activation of secondary metabolism in *Streptomyces*.

## Introduction

**Figure 1:**
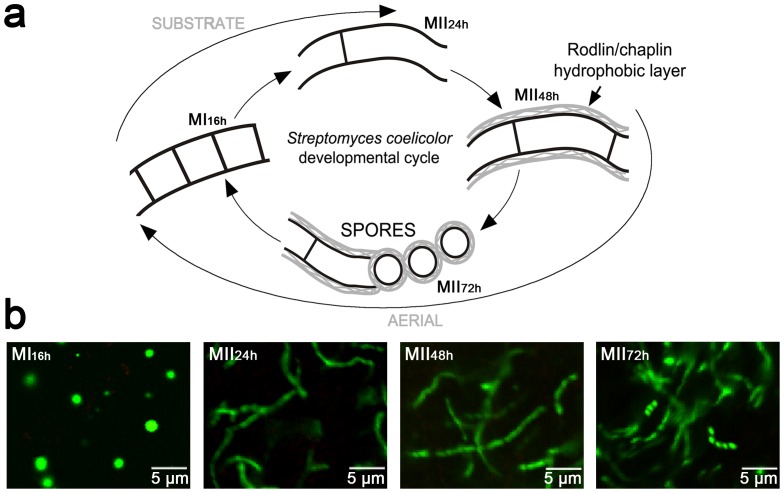
S. coelicolor development stages and sample preparation. (a) Cell-cycle features of Streptomyces development. Mycelial structures (MI, first compartmentalized mycelium; MII, second multinucleated mycelium). The classical nomenclature of substrate and aerial mycelium, and hydrophobic layers are indicated. (b) Confocal laser fluorescence micrographs of the different mycelia after fractioning and stained with SYTO9 and PI (see Methods). Notice the absence of dead cells (red staining). Mycelial types and developmental time points are indicated. See text for details.

*Streptomyces* is a very important industrial bacterium, which produces two thirds of all clinically relevant secondary metabolites. It is considered a “multicellular” prokaryotic model that includes programmed cell death (PCD) and sporulation. The classical *Streptomyces* developmental model for confluent solid cultures assumed that differentiation takes place along the transversal axis of the cultures (bottom-up): completely viable vegetative mycelia (substrate) grow on the surface and inside agar until they undergo a PCD, followed by hyphae differentiation into a reproductive (aerial) mycelium characterized by the presence of hydrophobic covers. Substrate and aerial mycelia are multinucleated, but at the end of the cycle, aerial hyphae form septa and spore chains (Figure 1a) (reviewed in Flärdh and Buttner [1]). Our research group has furthered our understanding of this developmental cycle, describing specific events that take place during the pre-sporulation stages (the phases preceding aerial mycelium formation and sporulation) [2]–[6]. We have characterized the existence of a previously unidentified, compartmentalized mycelium (MI) that initiates the developmental cycle following spore germination [2]–[6]. MI undergoes a highly ordered PCD [2], and the remaining viable segments of these hyphae begin to enlarge in the form of a multinucleated mycelium (MII). The traditionally denominated “substrate mycelium” corresponded to MII lacking hydrophobic layers, and the aerial mycelium to MII coated with these layers (Figure 1). MII has been demonstrated to be the antibiotic-producing mycelium [3].

**Figure 2:**
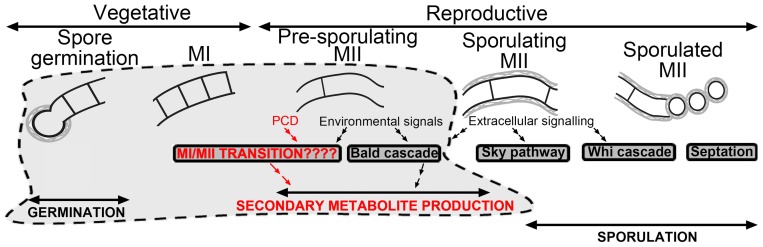
Schematic representation of biochemical pathways regulating Streptomyces differentiation. Pathways involved in hydrophobic covers formation (“bld”, “sky”), sporulation (“whi”, “septation”) are illustrated. Pre-sporulation pathways (“MI/MII transition”) not contemplated in the classical developmental model are labelled in red. See text for details.

Genetic studies of *Streptomyces* development regulation focused largely on the sporulation phases in solid cultures. *S. coelicolor* mutant strains defective in different stages of sporulation were used for the genetic and biochemical analyses of differentiation: the so-called “bald” (*bld*) mutants (considered defective in aerial growth) are affected in genes that regulate the so-called “sky-pathway” and activate the expression of genes encoding proteins forming hydrophobic covers (*rdl*, *chp*, *ram*); subsequently, the “white” (*whi*) genes (whose mutants are defective in the formation of mature grey spores on the tips of the white, fluffy aerial mycelium) are activated; finally, there is hyphae septation and sporulation (reviewed in Flärdh and Buttner [1] and Claessen et al [7]) (Figure 2).

In contrast to aerial mycelium and sporulation, *Streptomyces* pre-sporulation stages (germination and MI/MII transition) had been poorly studied (Figure 2). Spore germination was proven to include a succession of distinctive steps [8], [9]. Hardisson et al [9] organized these steps nicely into three stages: darkening, swelling and germ tube emergence. Darkening merely requires the presence of exogenous divalent cations (Ca^2+^, Mg^2+^ or Fe^2+^) with energy being obtained from spore reserves; swelling, needs an exogenous carbon source, and germ tube emergence requires additional carbon and nitrogen sources. Other works have demonstrated that germination is biochemically regulated: Guijarro et al [10] revealed the existence of a protein fraction inside the spores, which are rapidly degraded during germination and may participate in regulating this process; Mikulík et al [11] evidenced that RNA and protein synthesis begins in the first 5 min after germination, a fact that has recently been confirmed by Strakova et al [12]; Gund and Ensign [13] discovered the existence of germination inhibitors excreted in germination spores; Piette et al [14] established that the cyclic AMP receptor protein (crp) is involved in regulating spore germination; NepA was described as a structural cell wall protein that participates in maintaining spore dormancy [15]; Noens et al [16] identified SsgA as a protein that marks sites on the cell walls where germination taken place. Despite of all these studies, much more is still needed if we are to create an integrative model describing spore germination (Figure 2).

The molecular pathways regulating MI/MII transition and their involvement in secondary metabolism activation have scantly been studied. Our research group is pioneer in this regard. In recent works, we have analyzed proteins that are differentially expressed during development using high throughput proteomics (LC-MS/MS) in solid [5] and liquid [6] cultures. In this work, we have continued those studies by examining gene expression during development using transcriptomics. Proteins and genes differentially expressed in MI and MII are candidates as regulators of the MI/MII transition. This knowledge will facilitate future research aimed at understanding the biochemical pathways regulating *Streptomyces* pre-sporulation developmental stages and the activation of secondary metabolism (Figure 2).

## Materials and Methods

### Bacterial strains and media

The M145 strain of *Streptomyces coelicolor* was used in this study. Petri dishes (8.5 cm) with 25 ml of solid GYM medium (glucose, yeast/malt extract) [17] were covered with cellophane disks, inoculated with 100 µl of a spore suspension (1×10^7^ viable spores/ml), and incubated at 30°C. This medium promotes the rapid development of a lawn that differentiates readily and yields abundant sporulation.

### Sampling and fractioning of *Streptomyces coelicolor* cells throughout the differentiation cycle

The mycelium lawn of *S. coelicolor* M145 grown on cellophane disks was scraped off at different time points (16, 24, 48 and 72 hours) using a plain spatula. The samples were obtained at the 16-hour time point corresponded to MI; 24-hour samples to MII without hydrophobic covers (substrate); 48-hour sample to MII with hydrophobic covers (aerial); 72-hour sample to sporulating MII (Figure 1). Three independent cultures were prepared. At 16 hours, the first compartmentalized mycelium was separated from the non-septated mycelium by conversion of the cell compartments to protoplast forms [5], [6]. Lysozyme treatment was conducted at 30°C and incubation time was reduced to 5 minutes. Samples were washed twice by centrifugation for 5 minutes at 1000 g and 2 °C (to halt cell metabolism). Later, samples were preserved in RNA Protect (Quiagen®) at −80°C. MII samples were obtained during phases in which MI had died. Mycelial pellets were mechanically disaggregated (vigorous vortexing for 1 minute) in A buffer (Tris-HCl 50 mM pH 7, 150 mM NaCl, 10 mM MgCl_2_, EDTA 1 mM, ß-mercaptoethanol 7 mM, and protease inhibitor cocktail tablets, complete mini from Roche ref. 04693124001) (2.5 g of mycelium in 10 ml) pre-cooled to 0°C (to halt cell metabolism), and centrifuged for 5 minutes at 5000 g and 2°C. Mechanical disaggregation and washing steps were repeated 8 times. Samples were observed under confocal laser-scanning fluorescent microscopy after staining with vital dyes, as detailed below. Samples were preserved in RNA Protect (Quiagen®) at −80°C.

Four controls were designed to determine if mycelial fractioning induced significant changes in the transcriptional pattern (Figure S1). All controls were performed on the 24 hour sample. For the first control, the sample was collected as quickly as possible, immediately treated with *RNA Protect*, and frozen promptly; for the second control, MII was fractioned as described above before *RNA Protect* addition; for the third one, the conditions reached under the protoplasting process were simulated (incubation at 30°C for 5 minutes with lysozyme and kept in ice for 15 minutes, mimicking the washing steps after protoplast formation), prior to adding *RNA Protect*, and finally, for the last control, RNA protect was added before cellular fractioning.

### Viability staining

The permeability assay previously described for *Streptomyces* was used to stain the cultures [2]. The samples were observed under a Leica TCS-SP2-AOBS confocal laser-scanning microscope at a wavelength of 488 nm and 568 nm excitation and 530 nm (green) or 640 nm (red) emissions.

### RNA isolation and microarray hybridization

Total RNA samples from 3 biological replicates of each condition (dead/live) were obtained using phenol extraction and the *RNeasy Midi Kit* (Qiagen). RNA integrity was verified by means of the 2100 Bioanalyzer (Agilent). cDNA samples were synthesized and labelled using random hexamers, *SuperScript* III reverse transcriptase (Invitrogen), and Cy3-dCTP (GE Healthcare Life Sciences). Remnant RNA was hydrolyzed with NaOH and retrotranscription products were purified using the *MinElute PCR Purification Kit* (Qiagen). Genomic DNA from *S. coelicolor* M145 was used as the common reference. gDNA was labelled with Cy5-dCTP (GE Healthcare Life Sciences) using the *BioPrime Array CGH Genomic Labeling Module* (Invitrogen) and purified with the *MinElute* kit. Labelling efficiencies were quantified with a *NanoDrop* ND-1000 spectrophotometer. Mixtures of Cy3-cDNA (825 ng)/Cy5-gDNA (20 pmol of Cy5) were prepared in 110 µl of hybridization buffer (1 M NaCl, 100 mM MES, pH 6.5, 20% formamide, 20 mM EDTA, 1% Triton X-100). The microarrays for gene expression analysis were obtained from Oxford Gene Technology in the 4×44k format (Agilent *ink-jet* technology). A slide of this format comprises 4 identical matrices of 43,798 experimentally validated probes (60-mer oligonucleotides), which cover open reading frames (ORFs) and intergenic regions of the *S. coelicolor* genome [18]. The hybridization mixes were applied on the microarray surface following the manufacturer's instructions (100 µl), and hybridized at 55°C for 67 hours. The slide was washed in 50 ml of Agilent *Gene Expression Wash Buffer 1* for 5 min at room temperature, and then in 50 ml of *Wash Buffer 2* preheated at 37°C for 1 min. Both washing steps were carried out with horizontal agitation (85 rpm). The slide was then briefly immersed in the Agilent *Stabilization and Drying Solution* prior to fluorescence reading with an Agilent DNA Microarray Scanner G2565BA using the extended dynamic mode. Quantification of the fluorescence intensities were performed using the *FeatureExtraction* software (v9.5.1, Agilent).

### Transcriptome data analysis

Fluorescence intensities were processed using the R environment (R Development Core Team 2011, version 2.12.2) and the functions of the limma package [19]. For each spot on the microarray, net fluorescence intensities were calculated subtracting the median of background pixels values from the mean of foreground pixels. The standard deviation of the background pixels was used as the surrogate intensity when the subtraction was negative or lower than the background standard deviation. *S. coelicolor* genomic DNA labeled with Cy5 was used as the common reference. The Mg values were calculated as the log2 of Cy3-cDNA intensity divided by the Cy5-gDNA intensity. These values were normalized by first using cyclic loss (window of 0.3, 3 iterations) and then the median. For both normalizations, probe weights were used. BLAST comparisons enabled us to determine that 943 of the array probes had a potential cross-hybridization with more than one gene and that 7234 probes corresponded to intergenic regions. Weight values of 10^−6^ were assigned to these non-valid probes and 1 was the weight value assigned to the valid probes (35621). Values from valid probes of the same gene were averaged and limma linear models were used to obtain the Mc values of differential expression (log2 abundance values of comparisons between two conditions) and *p*-values, both FDR-corrected and uncorrected for multiple testing.

### Computational and bioinformatics analyses

ProteinCenter 2.0 (Proxeon, Denmark) was used to analyze and categorize genes. Genes were classified into functional categories according to their annotated functions in the Gene Bank database, and by homology/functions according to the Gene Ontology, the Conserved Domain, the KEGG pathway and StrepDB databases.

### Real-Time Quantitative Reverse Transcription PCR (qRT-PCR)

RNAs used in real-time RT-PCR analysis were digested with the *TURBO DNA-free™* kit (Ambion) to remove possible DNA contamination traces, following the rigorous treatment indicated in the manufacturer's instructions. Briefly, 50 µl of RNA solution at 200 ng/µl, were mixed with 1 µl of DNase and 5.6 µl of 10× Buffer and incubated at 37°C for 30 minutes. After that, 1 µl of DNase was added and incubated for other 30 minutes. Finally, samples were mixed with 0.2 volumes of inactivation reagent, incubated 5 min at room temperature and recovered by centrifugation.

One µg of RNA was used as the template for cDNA synthesis using the *High-Capacity cDNA Reverse Transcription Kit* (Applied Biosystems) according to the manufacturer's specifications. Primers used for Real-time PCRs are indicated in Table 1. Real-time PCRs were run on an *ABI PRISM 7900 HT* thermocycler (Applied Biosystems). Reactions contained 2 µl of cDNAs diluted 1/2, 10 µl of SYBR Green PCR Master Mix (Applied Biosystems) and 300 nM of primers in a final volume of 20 µl. Two biological samples were analyzed by triplicate and control reactions with RNA and water as templates were conducted to verify the absence of DNA contamination and primer-dimers formation. The thermal profile was as follows: an initial stage at 50°C for 2 min, a second incubation at 95°C for 10 min, a third stage of 40 cycles at 95°C for 15 s and 60°C for 1 min, and a final dissociation step at 95°C for 15 s, 60°C for 15 s and 95°C for 15 s to confirm the absence of primers dimers. Relative quantification of gene expression was performed by the ΔΔCt method [20]. SCO3878, encoding a β-chain of DNA polymerase III, was used as an internal control to quantify the relative expression of the target genes, since its expression level remained constant in all the conditions analyzed by microarray (Figure S2).

**Table 1:** Primers used for qRT-PCR.

Name	Sequence (5′–3′)	Target
SCO5898F	cggagaacaagggcaagc	*redF*
SCO5898R	Cagggggatggcgaag	*redF*
SCO7014F	gctcaggtggcgaagaag	SCO7014
SCO7014R	Gcaactcgggcaggac	SCO7014
SCO5085F	gcggctttttggaatgc	*actII4*
SCO5085R	gcagggtctcgttcagc	*actII4*
SCO6992F	cggaccttccacaacatcc	*absR1*
SCO6992R	tcgggctccagtatcagg	*absR1*
SCO5582F	ctcggctcctacatcctctc	*nsdA*
SCO5582R	ctccatcgcgtacagcatc	*nsdA*
SCO3878F	Gggcgtgctcatcctg	SCO3878
SCO3878R	gcttcgtggaggtcgtg	SCO3878

## Results

### Fractioning of *S. coelicolor* developmental stages (MI and MII)

As introduced above, *Streptomyces* is a mycelial bacterium with a complex developmental cycle which hinders its fractionation at distinctive developmental stages. We have recently developed a methodology to overcome this impediment in proteomic experiments [5]. In the present study, we adapted our methodology to separate RNA from MI and MII (Figure 1b). MI was obtained at 16 hours, MII without hydrophobic covers (substrate) at 24 hours, MII with hydrophobic covers (aerial) at 48 hours, and sporulating MII at 72 hours. MI was separated from the non-septated mycelium and dead hyphae by conversion of cell compartments to protoplasts as previously described, taking advantage of the fact that dying cells and multinucleated hyphae cannot form stable protoplasts [5], [6]. MII samples were obtained combining mechanical disaggregation of mycelial pellets with intensive washes, in order to remove the RNA released by the lysis of the MI (Figure 1b). Four controls were designed to determine whether the mycelial fractioning was modifying the transcriptional pattern in any way (see Methods for details) (Figure S1). In the first control, the sample was collected without fractionation, and treated with the protective RNA reagent (*RNA Protect*) as soon as possible and instantly frozen. In the second one, MII was fractionated as previously described above prior to *RNA Protect* addition. The third control was prepared in the following way: conditions reached during the protoplasting process (incubation at 30°C for 5 minutes with lysozyme and kept in ice for 15 minutes, mimicking the washing steps after protoplast formation) were simulated before *RNA Protect* was added. Finally, *RNA Protect* was added to the fourth control before cellular fractioning. Controls 1, 2 and 3 showed good correlation among them (correlation coefficients of 0.98) (Figure S1a) greater, in fact, than the correlations observed among biological replicates (correlation coefficient of 0.976) (Figure S1b) or at different developmental stages (Figure S1c). *RNA Protect* could not be added to stabilize RNA prior to cell fractioning (fourth control) because *RNA Protect* completely destroyed cell membrane permeability (data not shown) and RNA were modified tremendously during cellular fractionation (correlation coefficient with respect to first control of 0.93; data not shown). In order to prevent contamination between the RNA belonging to MI and that of MII, and get as close as possible to MI and MII transcriptomes, we decided to use the cellular fractioning protocol described above to separate MI and MII, accepting the variability that might be generated during mycelium fractioning, which was demonstrated to be minimal (second control) (Figure S1a).

### Global quantification of gene transcriptions

**Figure 3:**
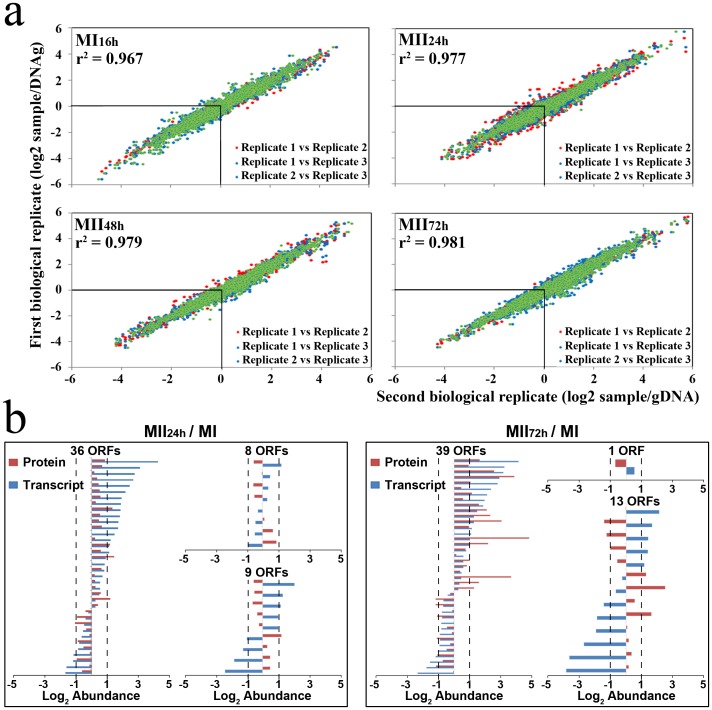
Quantitative transcriptomic data analysis. (a) Correlation of transcription abundance values (log2 ratio against chromosomal DNA) for biological replicates (three biological replicates compared in pairs) significantly quantified (*p*-value<0.05) (Table S1). Coefficients of regressions among replicates are shown. (b) Abundance values of the ORFs identified by proteomics [5] and transcriptomics (this work). Dashed lines indicate the limit for considering abundance variations as significant (log2 abundances greater than ±1).

**Figure 4:**
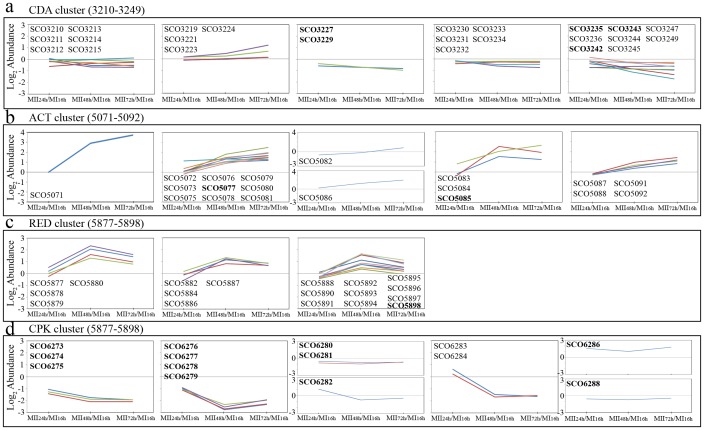
Quantitative transcriptomic data of genes involved in the synthesis of the most important secondary metabolites produced by *S. coelicolor*. (a) CDA cluster, genes SCO3210–SCO3249. (b) ACT cluster, genes SCO5071–SCO5092. (c) RED cluster, genes SCO5877–5898. (d) Cpk cluster, genes SCO6273–SCO6288. Genes were grouped into graphs according to their predicted operons. Genes included in the most reliable transcripts (Figure 3 and Table S1) are highlighted in bold. The remaining transcripts (Roman letters) also had good reproducibility in their abundance values, albeit not enough to meet the rigorous criteria used in this work (Table S2).

Three independent biological replicates and four developmental stages (MI_16 h_, MII_24 h_, MII_48 h_, MII_72 h_) were processed (12 samples in total) (Figure S3). With the aim of focusing on the most reliable data possible, we followed a very conservative strategy, selecting only genes with highly significant abundance values (*p*-values<0.05) for all the comparisons (MII_24 h_/MI_16 h_, MII_48 h_/MI_16 h_, MII_72 h_/MI_16 h_), and discarding genes with *p*-values>0.05 in at least in one of the comparisons analyzed (see Methods for details). We selected 1901 transcripts (24% of all *S. coelicolor* ORFs) with significant abundance values whose reproducibility is illustrated in Figure 3a. Several other transcripts (Table S1) also had adequate reproducibility, however, with the exception of the transcripts involved in secondary metabolite biosynthesis shown in Figure 4 (see below), they were not considered in the figures and discussions because the aim of this work was to characterize the most important and reliable differences between MI and MII transcriptomes instead to covering the entire *Streptomyces* transcriptome.

Fifty-three of the proteins encoded by these 1901 transcripts were identified in previous proteomic works that have addressed the analysis of MI and MII proteomes [5]. Correlation among protein and transcript abundances was very reasonable (Figure 3b), considering that they are totally different biomolecules with different turnovers. Protein and transcript ratios of MII_24 h_ with respect to MI were similar in 36 ORFs; had no significant variations in 8 ORFs (ratios<+/− 1); and only 9 ORFs showed different abundances when we analyzed proteins or transcripts (Figure 3b). In the case of MII_72 h_ with respect to MI, 39 ORFs had similar ratios; 1 ORF displayed no significant variations; and only 13 ORFs showed different abundance values (Figure 3b). The good correlation between protein and transcript abundances, together with the controls carried out demonstrating that the transcriptional pattern remained basically unaltered during sample processing (Figure S1), were an indicative of the reliability of the transcriptomic data obtained in this work.

### Similarities and differences between MI and MII transcriptomes: genes with well characterized functions

**Figure 5:**
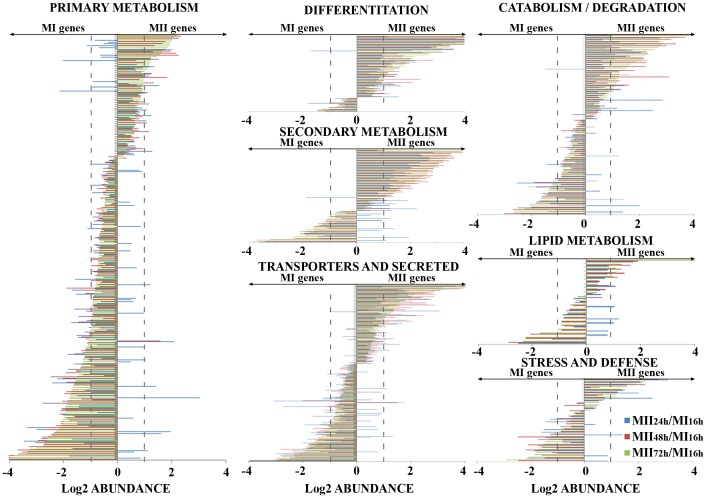
Abundance values (log2 MII/MI; averages from three biological replicates) of the genes significantly quantified (1901 in total) and grouped in functional categories. Primary metabolism (DNA/RNA replication, aerobic and anaerobic energy production, glycolysis and glyconeogenesis, pentose phosphate pathway, amino acid metabolism, nucleotide metabolism, translation, protein folding, RNA/protein processing, nucleases/RM methylases); secondary metabolism (secondary metabolites synthesis); differentiation (TTA BldA targets, Bld and Whi proteins); transporters and secreted (ABC transporters, transporters and secreted proteins); catabolism and degradation; lipid metabolism; stress and defense proteins. Dashed lines indicate the limit for considering abundance variations significant (log2 abundance ±1).

**Figure 6:**
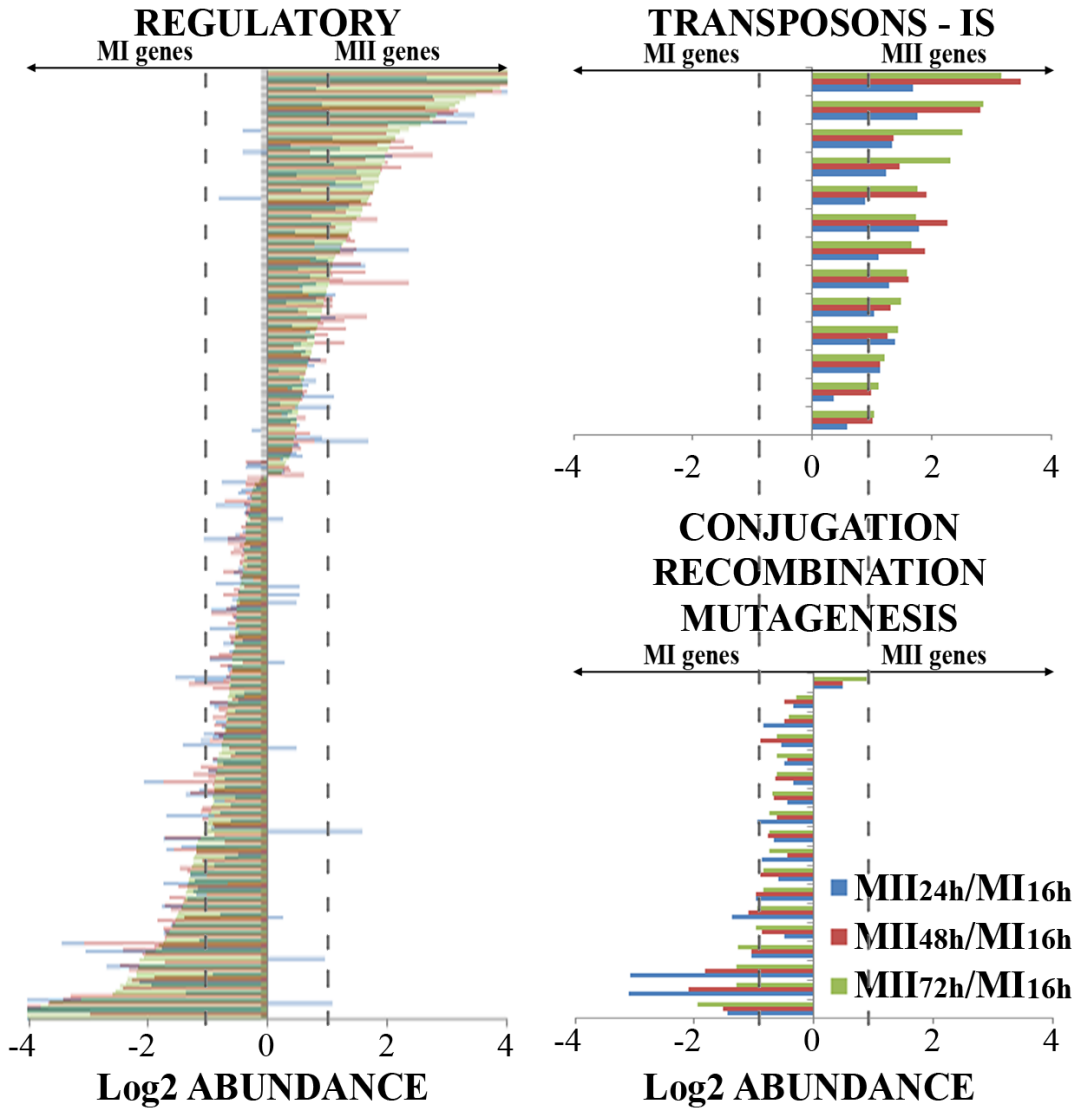
Gene abundance values (log2 MII/MI) of the genes grouped in functional categories (continuation). Regulatory proteins (transcriptional regulators, kinases, other regulatory proteins); transposons - insertion sequences; conjugation, recombination, mutagenesis. Dashed lines indicate the limit for considering abundance variations significant (log2 abundance ±1).

Next, we compared the MI and MII transcriptomes grouping the 1901 genes whose abundance was significantly quantified into functional categories (Figures 5 and 6). The MI stage served as reference, and transcript abundances were shown as the log2 ratio MII/MI (MII_24 h_/MI_16 h_, MII_48 h_/MI_16 h_ and MII_72 h_/MI_16 h_). Negative abundance values corresponded to genes up-regulated in MI, and the positive ones to genes up-regulated in MII. Most genes (95% of the total) had similar expression patterns in all the MII stages analyzed (MII_24 h_ – “substrate”; MII_48 h_, – “aerial”; MII_72 h_ – “spores”): the same genes were up- (positive abundance values in Figures 5 and 6) or down- (negative abundance values in Figures 5 and 6) regulated in MII with respect to MI.

Many well-characterized genes involved in primary metabolism were up-regulated in MI (Figure 5): up to 24-fold in the case of genes from oxidative phosphorylation (SCO0924, cytochrome B subunit; SCO3945, cytochrome oxidase *cydA*; SCO3946, cytochrome oxidase *cydB*); up to 6-fold in the case of genes encoding proteins involved in glycolysis and glyconeogenesis (SCO1947 and SCO7511, glyceraldehyde 3-phosphate dehydrogenases), or up to 2.5-fold in the case of genes encoding ribosomal proteins (*rplI*, SCO3909), just to name a few examples (Table 2).

**Table 2:** Well characterized genes showing the greatest abundance differences between MI and MII (see Table S1).

FUNCTION	SCO NO.	DESCRIPTION	LOG 2 ABUNDANCE		
			MII_24H_/MI_16H_	MII_48H_/MI_16H_	MII_72H_/MI_16H_
**Krebs cycle and energy metabolism**	SCO0924	Cytochrome B subunit	0.6	**−3.5**	**−3.3**
	SCO3945	Cytochrome oxidase subunit I, CydA	**−1.8**	**−4.6**	**−4.2**
	SCO3946	Cytochrome oxidase subunit II, CydB	**−1**	**−3.7**	**−3.5**
**Glycolysis and glyconoeogenesis**	SCO1947	Glyceraldehyde-3-phosphate dehydrogenase	0.6	**−1.9**	**−2**
	SCO7511	Glyceraldehyde-3-phosphate dehydrogenase	−0.9	**−2.7**	**−2.7**
**Ribosomal**	SCO3909	RplI	**−1.2**	**−1.3**	**−1.2**
**Hydrophobic covers – Sporulation**	SCO0409	SapA	**1**	**1.5**	**2.7**
	SCO1674	ChpC	**3.2**	**4.4**	**4.4**
	SCO1675	ChpH	**5.4**	**5.2**	**4.7**
	SCO1800	ChpE	**5.7**	**5.3**	**5.3**
	SCO2705	ChpF	0.8	**1.6**	**1.4**
	SCO2717	ChpD	**3.7**	**5.5**	**5.6**
	SCO2718	RdlA	**1.1**	**7**	**7.4**
	SCO3323	BldN	**4.6**	**4.3**	**4.2**
	SCO3579	WblA	**2.9**	**3.4**	**3.5**
	SCO4091	BldC	**1**	0.9	**1.4**
	SCO4543	WhiJ	**−1**	**−1.3**	**−1.4**
	SCO4768	BldM	**2.8**	**3**	**3.5**
	SCO5113	BldkB	**1.5**	0.9	0.6
	SCO5190	WblC	−1.7	**2**	**3.2**
	SCO5240	WblE	**2.2**	**1.4**	**2**
	SCO5316	WhiE	0.8	**1**	**3.9**
	SCO5319	WhiE protein II	**1.7**	0.9	**2.7**
	SCO5582	NdsA	**2.4**	**3.2**	**3.1**
	SCO5621	WhiG	0.4	0.9	0.6
	SCO5723	BldB	0.8	0.9	**1**
	SCO5819	WhiH	**1.3**	**2**	**1.8**
	SCO6681	RamC	0.8	**2.4**	**1.1**
	SCO6682	RamS	**3.7**	**5.3**	**4.6**
	SCO6683	RamA	0.9	**2**	**1**
	SCO6992	AbsR1	**1.5**	**4.7**	**5.8**
**Secondary metabolite synthesis**	SCO3227	Aminotransferase	−0.6	−0.7	−0.8
**(ACT, RED, CDA, CPK)**	SCO3229	4-hydroxyphenylpyruvic acid dioxygenase	−0.4	−0.7	−0.9
	SCO3235	ABC transporter	−0.4	−0.8	−0.9
	SCO3242	Putative transferase	−0.7	−0.8	−0.9
	SCO3243	Myo-inositol phosphate synthase	−0.7	−0.6	−0.6
	SCO5077	ActVA	**1.1**	**1.3**	**1.3**
	SCO5085	ActII-4	**1.4**	**2**	**2.6**
	SCO5898	RedF	**1.6**	**2.1**	**1.7**
	SCO6073	GeoA, cyclase	**2.2**	**1.2**	0.3
	SCO6273	Type I polyketide synthase cpkC	**−1**	**−1.4**	**−1.3**
	SCO6274	Type I polyketide synthase cpkB	**−1.7**	**−2.1**	**−1.9**
	SCO6275	Type I polyketide synthase cpkA	**−1.9**	**−2.1**	**−2.3**
	SCO6276	Secreted monooxygenase	−0.9	**−2.8**	**−2.3**
	SCO6277	Epoxide hydrolase	**−1.1**	**−2.7**	**−2.3**
	SCO6278	Integral membrane transport protein	**−1.1**	**−2.3**	**−1.9**
	SCO6279	Diaminobutyrate-pyruvate aminotransferase	−0.9	**−2.5**	**−1.9**
	SCO6280	Putative transcriptional regulator	−0.5	−0.7	−0.7
	SCO6281	FAD-binding protein	−0.8	−0.9	−0.6
	SCO6286	Putative transcriptional repressor	**1.6**	**1**	**1.8**
	SCO6288	Putative transcriptional regulator	−0.6	−0.7	−0.4

Average log2 abundance values from three biological replicates of the second multinucleated mycelial stages with respect to the first compartmentalized mycelium are shown. Significant abundant values (log2 abundance greater than ±1) are shown in bold. Functions (according to Gene bank, Gene Ontology, Conserved Domain, and KEGG and StrepDB databases).

Most of the well-characterized genes that participate in hyphal differentiation were up-regulated in MII (Figure 5): activators of aerial mycelium differentiation were up-regulated by up to 11-fold (*bldN*, SCO3323; *bldC*, SCO4091; *bldM*, SCO4768; *bldkB*, SCO5113; *bldB*, SCO5723); genes involved in the formation of hydrophobic covers were up-regulated by up to 169-fold (*sapA* spore-associated protein, SCO0409; *chpC*, SCO1674; *chpH*, SCO1675; *chpE*, SCO1800; *chpF*, SCO2705; *chpD*, SCO2717; *rdlA*, SCO2718; *ramC*, SCO6681; *ramS*, SCO6682; *ramA*, SCO6683); sporulation regulatory genes were up-regulated up to 13.5-fold (*wblA*, SCO3579; *wblC*, SCO5190; *wblE*, SCO5240; *whiE*, SCO5316; *whiE* protein II, SCO5319; *whiG*, SCO5621; *whiH*, SCO5819). As discussed below, *whiJ* (SCO4543) was the only exception as it was up-regulated in MI (by up to 2.7-fold).

*S. coelicolor* chromosome produces at least 4 different antibiotics [21]: actinorhodin (ACT cluster; genes SCO5071-SCO5092), prodigiosines (RED cluster; genes SCO5877–5898), calcium-dependent antibiotic (CDA cluster; genes SCO3210-SCO3249) and a yellow pigment with antibacterial activity (Cpk cluster; genes SCO6273–SCO6288) [21]–[23] whose structure has been recently elucidated [24]. The transcription of genes belonging to all these clusters was detected in this work (Table 2). Genes from the ACT cluster were up-regulated by up to 6-fold in MII (*actVA*, SCO5077; *actII-4*, SCO5085). *RedF* (SCO5898), a gene belonging to the prodigiosines cluster, was up-regulated by up to 4.3-fold in MII. Genes from the CDA cluster (SCO3227, SCO3229, SCO3235, SCO3242, SCO3243) exhibited no significant variations between MI and MII (relative log2 abundances MII/MI<±1). Variations of several genes belonging to the Cpk cluster were also detected (SCO6273, SCO6274, SCO6275, SCO6276, SCO6277, SCO6278, SCO6279, SCO6280, SCO6281, SCO6286, SCO6288). Most of these *Cpk* genes were up-regulated in MI (by up to 6.5-fold), with the exceptions of SCO6286 (a repressor that was up-regulated by 3.5-fold in MII), and SCO6280–SCO6281 which revealed not significant variations between MI and MII (log2 abundances<±1) (Table 2). Another secondary metabolite produced by *S. coelicolor* is the odorant geosmin [25]. The expression of one of the genes involved in its biosynthesis (*geoA*, SCO6073) was seen to be up-regulated in MII (by up to 4.6-fold at 24 hours) with respect to MI (Table 2). In addition to these genes included in the 1901 genes showing *p-values* less than 0.05 (Table S1), we identified more genes belonging to these secondary metabolite clusters with a pattern of expression that is consistent with differentiation (Table S2) (Figure 4). These transcripts had a good reproducibility within their respective operons (Figure 4) which was a further indication of the reliability of the transcriptomic data of this work.

Interestingly, all the genes encoding transposons and insertion sequences were revealed to be up-regulated in MII (Figure 6), suggesting an activation of these mobile genetic elements in the phases preceding sporulation. The opposite occurred with genes involved in conjugation, recombination, or mutagenesis, which were up-regulated in the MI phase, suggesting their activation in this stage, prior to the emergence of MII (Figure 6). The expression of genes related with transport and secretion, catabolism/degradation, lipid metabolism, stress-defense, and regulatory functions was not clearly biased toward MI or MII (Figures 5 and 6, Table S1).

### Similarities and differences between MI and MII transcriptomes: genes with unknown functions

In addition to the well-known developmental/metabolic genes described above, we were able to quantify the expression of several non-characterized genes differentially expressed during development (more than 4-fold up- or down- regulated in MI versus MII) (Table S1). These genes were reduced to 122 after filtering them, attending to conservation in the *Streptomyces* genus (similarity greater than 75% among *S. coelicolor*, *S. avermitillis* MA-4680, *S. griseus*, *S. clavuligerus* ATCC27064, and *S. scabies*) and to divergence in their expressions between MII/MI (log2 relative abundances>±2; genes up- or down-regulated by more than 4-fold in MII in comparison to MI) (Table S1). These genes included putative regulatory genes (transcriptional regulators, kinases, etc.), as well as genes encoding for proteins whose functions are unknown.

When we focused on those genes displaying the most differential expression (genes up- or down-regulated by more than 8-fold in MII with respect to MI), these 122 genes were reduced to 53 (Table 3). They encoded for 41 hypothetical proteins whose biological function could not be deduced by homologies, putative transcriptional regulators, putative sporulation proteins, and other regulatory proteins (Table 3).

**Table 3:** Most differentially expressed genes among MI and MII with unknown function and conserved in the *Streptomyces* genus.

Function	SCO no.	Description	Log 2 abundance		
			MII_24 h_/MI_16 h_	MII_48 h_/MI_16 h_	MII_72 h_/MI_16 h_
**Sporulation**	SCO2805	Putative sporulation protein	0.8	**3.5**	**3.4**
**DNA/RNA replication**	SCO5494	NAD-dependent DNA ligase LigA	**−3.4**	**−2.5**	**−2.2**
**Transcriptional Regulators**	SCO0944	Predicted transmembrane transcriptional regulator	**1**	**3.2**	**3**
	SCO5656	Transcriptional regulatory protein	**−4.1**	**−4.3**	**−3.8**
	SCO4640	TetR family transcriptional regulator	**−4.5**	**−4.5**	**−4.2**
	SCO7014	LacI family transcriptional regulator	**−2.5**	**−3**	**−4.5**
	SCO4032	MarR regulatory protein	**−4.3**	**−5.1**	**−5.4**
	SCO4336	MarR-family protein	**−2.1**	**−3.3**	**−2.6**
	SCO2730	TetR-family transcriptional regulator	**−3**	**−2.4**	**−1.9**
**Other Regulatory Proteins**	SCO5351	Regulatory protein	0.8	2.8	3.3
	SCO3134	Two-component system response regulator	0.9	3	3.2
	SCO1629	rarB homologue	**3.4**	**3.1**	**2.8**
**Unknown**	SCO5447	Neutral zinc metalloprotease	0.4	**3.1**	**1.2**
	SCO4440	Hypothetical protein	**1.6**	**4.6**	**4.9**
	SCO0682	Hypothetical protein	**4.3**	**4.5**	**4.7**
	SCO1756	hypothetical protein	**1.8**	**3.9**	**4.7**
	SCO2492	Hypothetical protein	**2.5**	**4.8**	**4.5**
	SCO0930	Putative lipoprotein	0.9	**4.4**	**4.3**
	SCO0683	Hypothetical protein	**3.8**	**4.4**	**4.2**
	SCO6165	Putative dnaK suppressor	**−1.2**	−0.9	**4.2**
	SCO3328	Hypothetical protein	**2.1**	**3.7**	**4.2**
	SCO6652	Hypothetical protein	0.8	**4.3**	**4.1**
	SCO7251	Hypothetical protein	**4.1**	**4.4**	**4.1**
	SCO1474	Hypothetical protein	**2.8**	**3.4**	**4**
	SCO1029	Hypothetical protein	**2**	**3.8**	**3.9**
	SCO2819	Hypothetical protein	**2.2**	**3.7**	**3.8**
	SCO0644	Possible membrane protein	0.6	**4**	**3.9**
	SCO4335	Hypothetical protein	**2.1**	**3.3**	**3.6**
	SCO5175	Hypothetical protein	**2.4**	**3.8**	**3.5**
	SCO5174	Transferase	**2.2**	**3.5**	**3.5**
	SCO3324	Hypothetical protein	**2.7**	**3.1**	**3.5**
	SCO5191	Hypothetical protein	**−2**	**2.1**	**3.3**
	SCO0684	Hypothetical protein	**2.7**	**3.4**	**3.3**
	SCO7253	Hypothetical protein	**1.2**	**3.1**	**3.2**
	SCO5834	Hypothetical protein membrane spanning	**1.5**	**3.1**	**3.2**
	SCO2302	Hypothetical protein	**2.6**	**3.1**	**3.2**
	SCO4173	Hypothetical protein	**2.5**	**2.8**	**3.1**
	SCO5177	Hypothetical protein	**2.6**	**3.3**	**3**
	SCO1860	Hypothetical protein	**2.7**	**3.4**	**3**
	SCO5655	Hypothetical protein	**−4**	**−4.4**	**−4.3**
	SCO5484	Small hydrophobic membrane protein	**−4.7**	**−5.2**	**−5.6**
	SCO1823	Hypothetical protein	**1.2**	**3.9**	**2.1**
	SCO0932	Hypothetical protein	**1.8**	**3.5**	**2.8**
	SCO6795	Hypothetical protein	**2.5**	**3.4**	**2.5**
	SCO5249	Nucleotide-binding protein	**2.9**	**3.2**	**2.4**
	SCO6797	ATP/GTP binding protein	**2.2**	**3**	**2.2**
	SCO2205	Hypothetical protein	**−3.5**	**−3.6**	**−1.9**
	SCO7471	Phenylacetate-CoA oxygenase PaaA	**3.4**	**1.2**	**1.3**
	SCO1105	Hypothetical protein	**3.2**	**1.8**	0.8
	SCO7472	Phenylacetate-CoA oxygenase PaaB	**3.2**	0.8	0.7
	SCO4793	Probable NPL/P60 family secreted protein	**1.7**	**3.8**	**3.9**
	SCO7453	Putative secreted protein	**1.5**	**1.7**	**3.4**
	SCO4677	Histidine kinase-like ATPase	**4.2**	**4.4**	**4.1**

Average log2 abundance values and functions as in Table 2. Genes whose function was not characterized, with average log2 relative abundances greater than ±3 in at least one of the developmental phases analyzed (up- or down- regulated by greater than 8-fold in MII at 24, 48 or 72 hours with respect to MI) are shown (see text for details). Only genes with conservation (similarity)>75% among *S. coelicolor*, *S. avermitillis* MA-4680, *S. griseus*, *S. clavuligerus* ATCC27064 and *S. scabies* were selected.

### Validation of microarray results using qRT-PCR

Finally, and in order to validate our quantitative data, we compared the abundances of some of the transcripts quantified by microarrays with abundances obtained by qRT-PCR. We analyzed the expression of the most differentially expressed genes (SCO6992, *absR1* activator of secondary metabolism; SCO7014, putative *lacI* family transcriptional regulator); two genes involved in the synthesis of actinorhodin (*actII-4*, SCO5085) and undecylprodigiosin (*redF*, SCO5898), and a putative regulator of differentiation (*ndsA*, SCO5582). Gene expressions quantified with both methodologies were similar (Figure S1): SCO6962, *actII-4*, *redF* and *ndsA* were up-regulated in MII at 24, 48 and 72 hours in comparison with MI (positive log2 abundance values); SCO7014 was up-regulated in MI (negative log2 abundance values). The correlation between transcriptomics and qRT-PCR was adequate (regression coefficient of 0.8) (Figure S2).

## Discussion

Several *Streptomyces* transcriptomic works have described genetic expressions at different developmental time points in liquid cultures [26], [27], at particular time points in solid cultures [28], or have compared *Streptomyces* mutants with the wild type strain in liquid [29] or solid [15], [30] cultures. However, this work is the first one that has focused specifically on analysing the differences between MI and MII transcriptomes in *S. coelicolor* wild type strain. We performed cellular fractionation so as to avoid contamination between MI and MII transcriptomes insofar as possible (Figure 1). Moreover, we focused on the most consistent data, reporting the variation in abundance of 1901 transcripts in MI and MII (Figure 3). These genes constituted a small subset of *Streptomyces* transcriptome (24% of all *S. coelicolor* ORFs), however, they represented the most reliable differences between MI and MII transcriptomes, which was the main objective of this work.

The expression of well characterized key developmental genes correlated well with the state of the art of what we know about *Streptomyces* differentiation (reviewed in Flärdh and Buttner [1] or Claessen et al [7], for instance), and therefore corroborating the reliability of the data presented here: genes involved in aerial mycelium differentiation (*bld*), in the formation of hydrophobic covers (*rdl, chp, ramS*), or sporulation (*wbl* and *whi* genes), were up-regulated in the differentiated MII; genes involved in actinorhodin, undecylprodigiosin, or geosmin biosynthesis were activated during the MII phase (Table 2, Figure 4, Table S1). Under the growth conditions used in this work, there was no CDA or Cpk production (data not shown) and the genes involved in their biosynthesis were not expressed (Table 2, Table S1). Interestingly, new insight into these developmental/metabolic genes was also obtained. *WhiJ* (SCO4543) was recently characterized as a repressor of sporulation [31], and we have discovered here that its transcription was more abundant (by up to 2.7-fold) (Table 2) during the non-sporulation phase (MI). *SarA* (SCO4543) was also described as a repressor of sporulation [32], and its transcription was also up-regulated in MI (by up to 1.7-fold) (Table S1, not included into the most differentially expressed genes of Table 2). Cpk production was shown to be repressed in culture media containing glucose [23]; however, the genetic regulation of this repression has remained uncharacterized. We reported high expression of SCO6286, a putative transcriptional regulator with homologies with transcriptional repressors (according to Conserved Domain Database) in MI (Table 2); this could be repressing the expression of the remaining Cpk cluster genes in our glucose-containing culture medium.

In addition to the five well characterized clusters involved in secondary metabolism described above, in silico analyses of the *S. coelicolor* genome have revealed the existence of another 15 clusters of genes encoding putative secondary metabolites [21]. The expression of genes belonging to 7 of these clusters has been quantified in this work (Table 4). Most of these genes were up-regulated in MII (Table 4). The only exceptions were genes forming the entire cluster of isorenieratene (SCO0185–SCO0191), and SCO0490, one esterase belonging to the putative coelichelin cluster, which were up-regulated in MI with respect to MII (Table 4). These results might be indicating that production of these secondary metabolites was repressed in the culture conditions used or even, that they might be produced during the vegetative stage (MI).

**Table 4:** Genes putatively involved in secondary metabolism (according to Bentley et al [21]) (see Table S1 for details).

SCO no.	Description	Log 2 abundance		
		MII_24 h_/MI_16 h_	MII_48 h_/MI_16 h_	MII_72 h_/MI_16 h_
SCO0185	Geranylgeranyl pyrophosphate synthase	−0.8	−0.9	**−1**
SCO0186	Phytoene dehydrogenase	**−1.2**	**−1.6**	**−1.6**
SCO0187	Phytoene synthase	−0.7	−0.6	−0.5
SCO0188	Methylesterase	**−1.3**	**−1.4**	**−1.3**
SCO0189	Dehydrogenase	−0.8	**−1.1**	**−1.2**
SCO0190	Methyltransferase	**−1.4**	**−2.3**	**−2.4**
SCO0191	Lycopene cyclase	**−1.2**	**−1.9**	**−2.1**
SCO0381	Glycosyl transferase	**2.4**	**3.5**	**3.6**
SCO0382	Dehydrogenase	**1.9**	**3.1**	**3.1**
SCO0383	Hypothetical protein	**2.9**	**4**	**3.8**
SCO0384	Putative exopolysaccharide transport	**1.8**	**2.8**	**2.6**
SCO0385	Putative exopolysaccharide transport	**1.8**	**2.8**	**2.7**
SCO0386	Asparagine synthetase	**1.2**	**2.3**	**2.2**
SCO0387	Bi-domain-containing oxidoreductase	**2**	**3.1**	**2.9**
SCO0388	Hypothetical protein	0.8	**1.7**	**1.5**
SCO0389	Lipoprotein	**1.4**	**2.8**	**2.7**
SCO0390	Hypothetical protein	**1.4**	**2.7**	**2.5**
SCO0391	Transferase	**1.2**	**2.6**	**2.3**
SCO0392	Methyltransferase	**2**	**2.9**	**2.9**
SCO0393	Transferase	**2.4**	**3.5**	**3.5**
SCO0394	Hypothetical protein	**2.4**	**3.3**	**3.2**
SCO0395	Epimerase/dehydratase	**2.7**	**3.4**	**3.2**
SCO0396	Putative Peptidase	**2.7**	**3.7**	**3.4**
SCO0397	Hypothetical protein	**1.2**	**2.1**	**1.7**
SCO0398	Glycosyl transferase	**1.8**	**2.6**	**2.2**
SCO0399	Possible membrane protein.	**2.7**	**3.6**	**3.3**
SCO0400	Epimerase	**2.3**	**3.3**	**2.9**
SCO0401	Glutamate-1-semialdehyde 2,1-aminomutase	**1.5**	**2.4**	**2.2**
SCO0490	Esterase	0.9	**−1.2**	**−1.1**
SCO0491	ABC transporter transmembrane protein	0.9	−0.4	−0.8
SCO0492	Peptide synthetase	**1.2**	−0.6	**−1.1**
SCO0493	ABC-transporter transmembrane protein	0.5	−0.8	−0.9
SCO0497	Iron-siderophore permease	0.8	−1.6	−1.8
SCO0498	Peptide monooxygenase	**2**	**−1.4**	**−2.3**
SCO0499	Methionyl-tRNA formyltransferase	**1.4**	**−1**	**−1.6**
SCO1267	Acyl carrier protein	**−3.6**	**−3.4**	**−3.9**
SCO2782	Pyridoxal-dependent decarboxylase	**1.1**	**−3.7**	**−3.2**
SCO5799	Aminotransferase	0.4	**1.3**	**1.8**
SCO5801	hypothetical protein	0.8	**1.8**	**1.3**
SCO6424	Histidine kinase	−0.4	0.7	**2.3**
SCO6435	Hypothetical protein	−0.4	−0.6	**−1.1**
SCO6760	Phytoene synthase	0.5	0.4	0.4
SCO6762	Phytoene dehydrogenase	0.9	**1.1**	**1.1**
SCO6763	Polyprenyl synthatase	**1**	**1**	0.9
SCO6764	Squalene-hopene cyclase	**1.4**	0.7	0.5
SCO6766	Hypothetical protein	**2.5**	**1.1**	0.9
SCO6767	4-hydroxy-3-methylbut-2-en-1-yl diphosphate synthase	**1.2**	0.6	0.5
SCO6768	Probable transketolase	**1.3**	0.5	0.2
SCO6769	Aminotransferase	**1.3**	0.4	0.3

Average log2 abundance values and functions as in Table 2.

Most of the genes identified (95% of the total) had similar expression patterns in all the MII phases analyzed (MII_24 h_ – “substrate”; MII_48 h_, - “aerial”; MII_72 h_ – “spores”): the same genes were up- (positive abundance values in Figures 5 and 6) or down- (negative abundance values in Figures 5 and 6) regulated in MII compared to MI. Overall, and despite the fact that activation of some secondary metabolite production such as Cpk required specific nutritional requirements (glucose) [23], secondary metabolism and hypha differentiation were activated in MII. The opposite happened with genes involved in primary metabolism which were up-regulated in MI (Figure 5). These results prove that MI and MII corresponded to different physiological stages. This is not a trivial result, as it demonstrates that all MII stages (substrate, aerial and sporulating mycelia) were very similar and differed from MI. These findings are consistent with proteomic data [5], [6] and morphological differentiation [3]: MI is the vegetative mycelium, and MII corresponds to the differentiated hyphae involved in secondary metabolism, which expressed genes involved in hydrophobic cover formation and sporulation beginning at time points as early as 24 hours (substrate mycelium).

In addition to the well-known developmental/metabolic genes described above, we were able to detect 122 genes differentially expressed in MI or MII (more than 4-fold), having no assigned biological function, but highly conserved in the *Streptomyces* genus (Table S1 and Table 3). These genes encoded putative regulatory proteins (transcriptional regulators, kinases), as well as hypothetical proteins, and their identification was the main goal of this work. Indicative of the importance of these genes and their potential to be involved in regulation of *Streptomyces* differentiation, is the fact that well characterized key developmental genes (*bld*, *whi*, *wbl*, *rdl*, *chp*, *ram* etc.) were also detected by the experimental approach used in this work. All this knowledge contributes to advance in the necessary work to understand the biochemical pathways controlling pre-sporulation developmental stages and the activation of secondary metabolism in *Streptomyces*.

## Supporting Information

Figure S1 **Overview of transcriptomic technical and biological reproducibilities.** (a) Effect of mycelial fractionation in the identified transcripts. Three controls were performed in the 24 hours sample: in the first control, the sample was collected without fractionation treated as quickly as possible with the protective RNA reagent (RNA protect), and frozen immediately (24 h – fractionation); in the second control, MII was fractioned combining mechanical disaggregation of mycelial pellets with intensive washes before RNA protect addition (24 h + fractionation) (see Methods for details); in the third one, conditions reached under the protoplasting process (incubation at 30°C for 5 minutes with lysozyme and kept in ice for 15 minutes, mimicking the washing steps after protoplast formation) were simulated before RNA protect addition (24 h + protoplasting). (b) Correlation between transcriptomes of two biological replicates obtained at 24 hours by mycelial fractioning. (c) Correlation between transcriptomes from different mycelial stages (MI_16 h_, MII_24 h_, MII_48 h_, MII_72 h_) obtained by myclial fractioning. Abundance values correspond to the average of three biological replicates indicated in Table S2. All the abundance values refer to chromosomal DNA (log_2_ sample/DNAg). Coefficients of regression among replicates are shown.
(TIF)

Figure S2 **Validation of microarray results using qRT-PCR.** (a) Correlation between microarray and qRT-PCR abundance values (log2 MII/MI versus log_2_ 2^ΔΔCt^) for 5 genes (see text for details). (b–f) Comparison of abundance values obtained by microarray and qRT-PCR for each gene. Values are the average and SD from two biological and three methodological replicates.
(TIF)

Figure S3 **Overview of the microarray strategy**. Three independent arrays (OGT 4×44k) were used for three independent biological replicates. Samples (MI_16 h_, MII_24 h_, MII_48 h_ and MII_72 h_) were labeled with Cy3; chromosomal *S. coelicolor* DNA was used as reference and labeled with Cy5.
(TIF)

Table S1 **Quantitative data of the expression of the most reliable** ***Streptomyces coelicolor*** **transcripts.** Relative transcript abundances are shown as the log2 ratio MII/MI. Only genes with significant abundances (*p*-values <0.05 in all the conditions) are represented: 1901 genes, those shown in Figures. 4, 5, and 6. Functional categories: 1. cell division/septation; 2. DNA/RNA replication; 3. TTA BldA targets and Bld Whi proteins; 4. transcriptional regulators; 5. other regulatory proteins; 6. Krebs cycle and energy metabolism; 7. catabolism, degradation; 8. glycolysis and glyconeogenesis; 9. pentose phosphate pathway; 10. amino acid metabolism; 11. lipid metabolism; 12. nucleotide metabolism; 13. other anabolic enzymes; 14. translation, protein folding, RNA/protein processing; 15. stress/defense proteins; chaperons/homeostasis; 16. unknown; 17. secondary metabolites synthesis; 18. nucleases/RM methylases; 19. ABC transporters; 20. transporters/secreted; 21. kinases/phosphatases; 22. DNA competence.
(XLSX)

Table S2 **Quantitative data of the expression of all** ***Streptomyces coelicolor*** **identified transcripts.** All transcriptomic data are shown, also including genes from Table S1. Mg values refer to chromosomal DNA. *P*-values are indicated.
(XLSX)
