# Supplementary figures and images for: Transcriptomic Analysis of Streptomyces coelicolor Differentiation in Solid Sporulating Cultures: First Compartmentalized and Second Multinucleated Mycelia Have Different and Distinctive Transcriptomes
Source: PLoS One. 2013 Mar 28;8(3):e60665. doi: 10.1371/journal.pone.0060665 (PMC3610822; doi:10.1371/journal.pone.0060665)

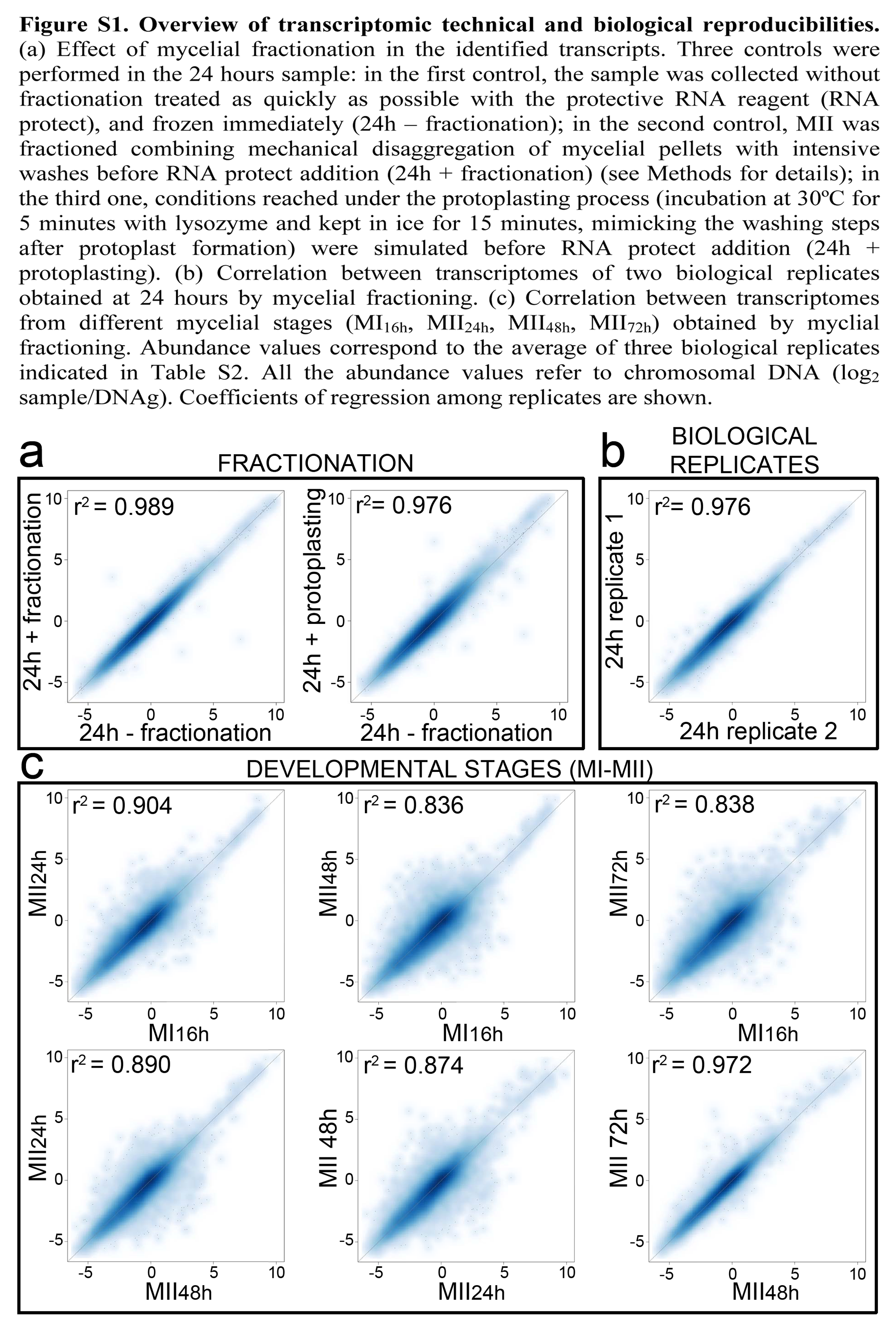

Supplement: Figure S1 — Overview of transcriptomic technical and biological reproducibilities. (a) Effect of mycelial fractionation in the identified transcripts. Three controls were performed in the 24 hours sample: in the first control, the sample was collected without fractionation treated as quickly as possible with the protective RNA reagent (RNA protect), and frozen immediately (24 h – fractionation); in the second control, MII was fractioned combining mechanical disaggregation of mycelial pellets with intensive washes before RNA protect addition (24 h + fractionation) (see Methods for details); in the third one, conditions reached under the protoplasting process (incubation at 30°C for 5 minutes with lysozyme and kept in ice for 15 minutes, mimicking the washing steps after protoplast formation) were simulated before RNA protect addition (24 h + protoplasting). (b) Correlation between transcriptomes of two biological replicates obtained at 24 hours by mycelial fractioning. (c) Correlation between transcriptomes from different mycelial stages (MI16 h, MII24 h, MII48 h, MII72 h) obtained by myclial fractioning. Abundance values correspond to the average of three biological replicates indicated in Table S2. All the abundance values refer to chromosomal DNA (log2 sample/DNAg). Coefficients of regression among replicates are shown. (TIF) [file pone.0060665.s001.tif]

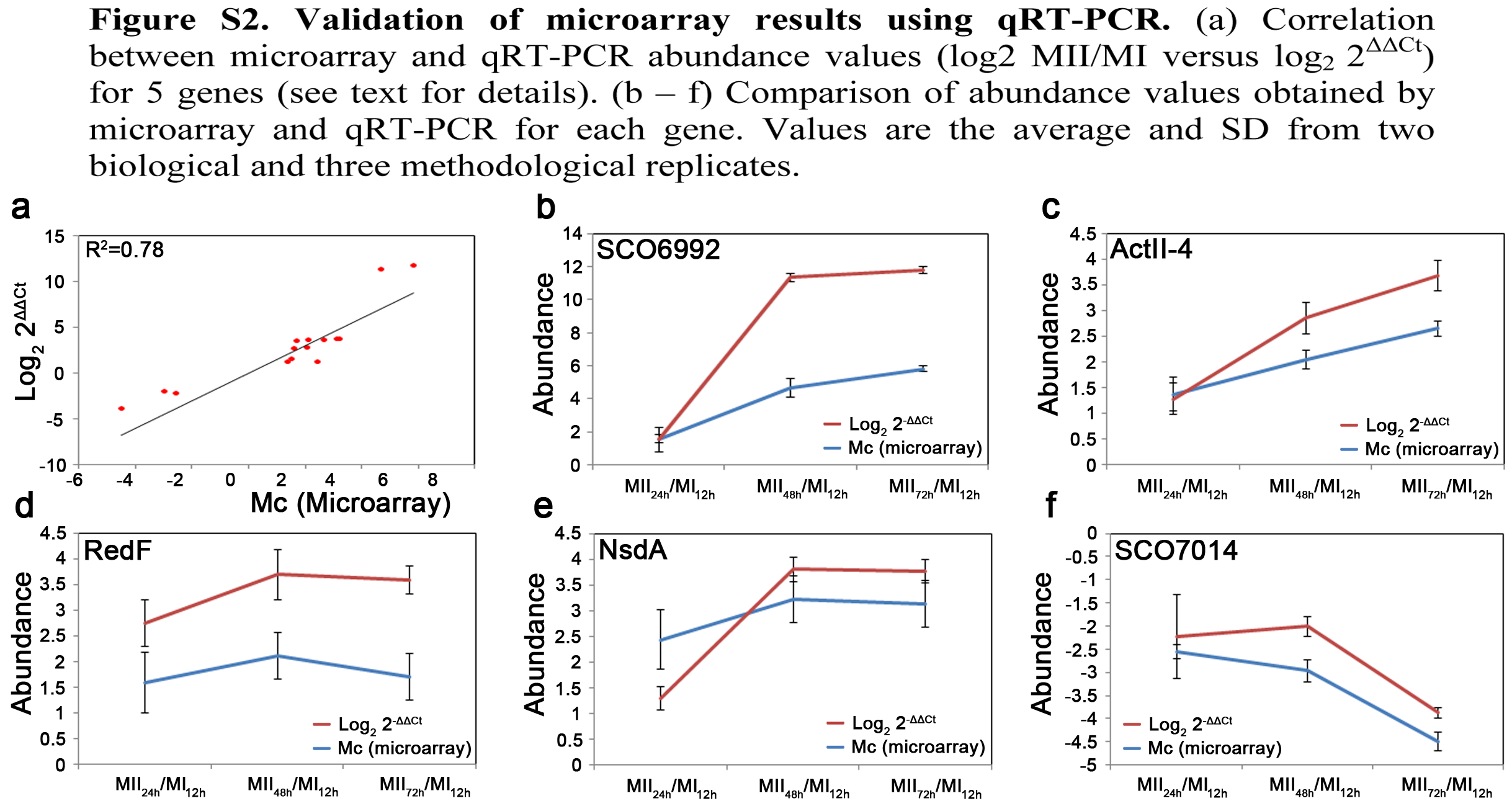

Supplement: Figure S2 — Validation of microarray results using qRT-PCR. (a) Correlation between microarray and qRT-PCR abundance values (log2 MII/MI versus log2 2ΔΔCt) for 5 genes (see text for details). (b–f) Comparison of abundance values obtained by microarray and qRT-PCR for each gene. Values are the average and SD from two biological and three methodological replicates. (TIF) [file pone.0060665.s002.tif]

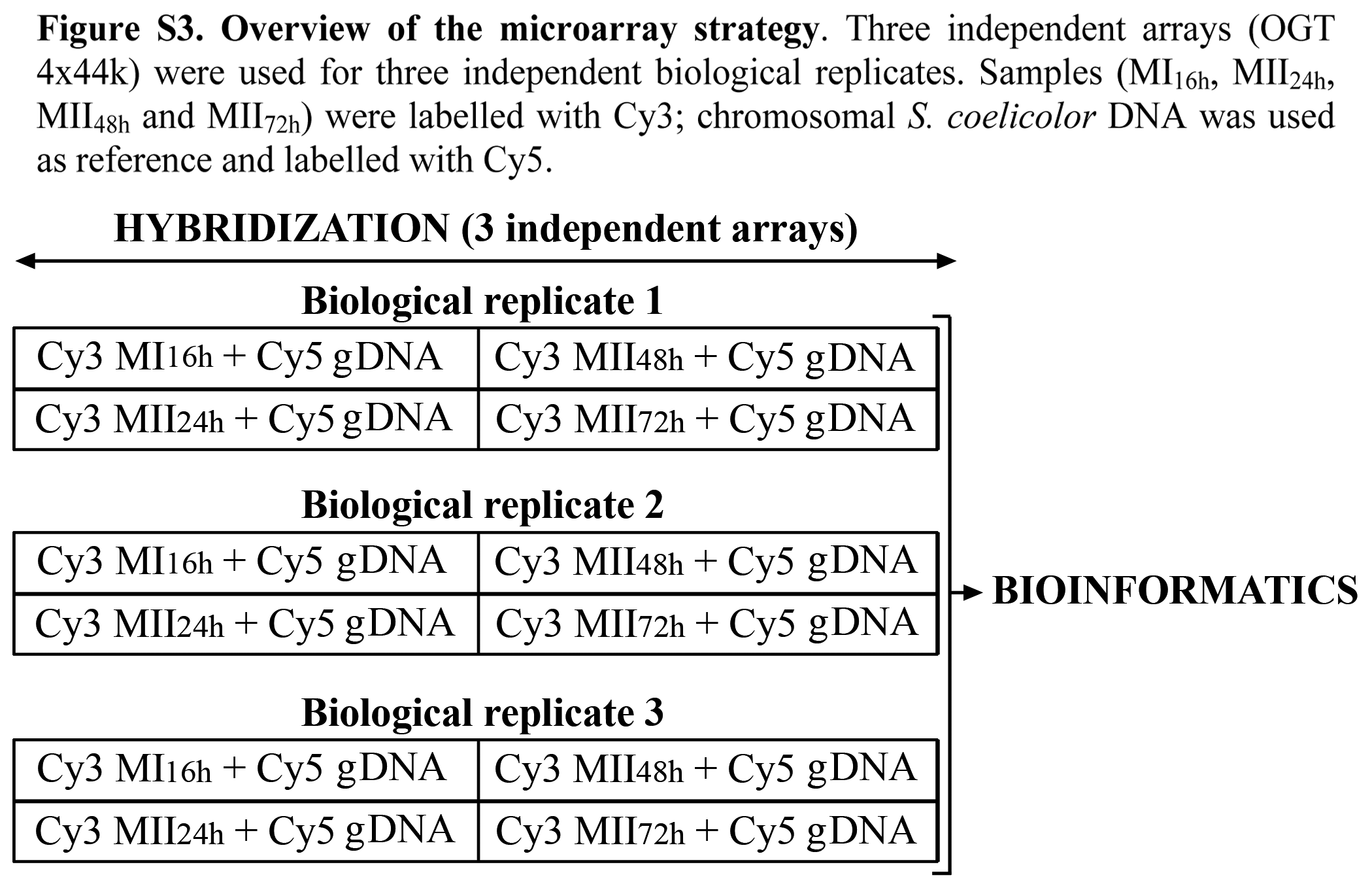

Supplement: Figure S3 — Overview of the microarray strategy. Three independent arrays (OGT 4×44k) were used for three independent biological replicates. Samples (MI16 h, MII24 h, MII48 h and MII72 h) were labeled with Cy3; chromosomal S. coelicolor DNA was used as reference and labeled with Cy5. (TIF) [file pone.0060665.s003.tif]
